# The Hypoxia-Mimetic Agent Cobalt Chloride Differently Affects Human Mesenchymal Stem Cells in Their Chondrogenic Potential

**DOI:** 10.1155/2018/3237253

**Published:** 2018-03-13

**Authors:** Gabriella Teti, Stefano Focaroli, Viviana Salvatore, Eleonora Mazzotti, Laura Ingra', Antonio Mazzotti, Mirella Falconi

**Affiliations:** ^1^Department of Biomedical and Neuromotor Sciences, University of Bologna, Bologna, Italy; ^2^An2H Discovery Limited, National Institute of Cellular Biotechnology (NICB), Dublin City University Campus, Dublin, Ireland; ^3^Faculty of Comparative Biomedical Sciences, University of Teramo, Teramo, Italy; ^4^First Orthopaedic and Traumatologic Clinic, Istituto Ortopedico Rizzoli, University of Bologna, Bologna, Italy

## Abstract

Adult stem cells are a promising cell source for cartilage regeneration. They resided in a special microenvironment known as the stem-cell niche, characterized by the presence of low oxygen concentration. Cobalt chloride (CoCl_2_) imitates hypoxia in vitro by stabilizing hypoxia-inducible factor-alpha (HIF-1*α*), which is the master regulator in the cellular adaptive response to hypoxia. In this study, the influence of CoCl_2_ on the chondrogenic potential of human MSCs, isolated from dental pulp, umbilical cord, and adipose tissue, was investigated. Cells were treated with concentrations of CoCl_2_ ranging from 50 to 400 *μ*M. Cell viability, HIF-1*α* protein synthesis, and the expression of the chondrogenic markers were analyzed. The results showed that the CoCl_2_ supplementation had no effect on cell viability, while the upregulation of chondrogenic markers such as SOX9, COL2A1, VCAN, and ACAN was dependent on the cellular source. This study shows that hypoxia, induced by CoCl_2_ treatment, can differently influence the behavior of MSCs, isolated from different sources, in their chondrogenic potential. These findings should be taken into consideration in the treatment of cartilage repair and regeneration based on stem cell therapies.

## 1. Introduction

Hyaline articular cartilage has a very limited or no intrinsic capacity for repair and minor traumatic lesions or pathological injuries may trigger progressive damage and joint degeneration [1]. Early intervention is needed to avoid growth of traumatic chondral and osteochondral defects and to delay cartilage degeneration and osteoarthritis.

Novel cell-based tissue engineering techniques have been proposed with the aim to repair cartilage defects and reconstitute the properties of hyaline cartilage. The mesenchymal stromal cells (MSCs), due to the high proliferative capacity, self-renewal, and potential to differentiate into different lineages, represent a promising strategy in regenerative medicine [2]. The MSCs are separated from several human tissues such as the bone marrow [3], the synovial tissue [4], the adipose tissue [5, 6], the periosteum [7, 8], the dental pulp [9] and Wharton's jelly umbilical cord [10].

The microenvironment of MSCs is characterized by a low oxygen tension, demonstrating that MSCs might be quite resistant to oxygen limitation [11]. Changes in the oxygen concentration activate intracellular mechanisms responsible either for cell death or for cell adaptation to new environmental conditions [11, 12]. The key adaptive response to hypoxic conditions is the stabilization of hypoxia-inducible factor- (HIF) 1 [13]. As a transcription factor, it plays a vital role in the functional expression of a number of genes involved in the adaptation and survival of cells, tissues, and organs. The transcription factor HIF-1 is composed of two subunits, HIF-1*α* and HIF-*β* or aryl hydrocarbon receptor nuclear translocator (ARNT) [14]. In both normoxic and hypoxic conditions, the HIF-*β* is constantly biosynthesised, degraded, and recycled [15]. HIF-1*α* in normal oxygen condition, despite being biosynthesised, is subjected to the instantaneous decomposition [16]. In normoxic conditions, HIF-1*α* is polyhydroxylated by oxygen-dependent prolyl (P4HS) and arginyl hydroxylases (FIH). Once hydroxylated, HIF-1*α* protein binds to von Hippel–Lindau (VHL) tumor suppressor protein—the recognition component of E3 ubiquitin-protein ligases—and is rapidly degraded by the proteasome. In hypoxic condition, hydroxylation is inhibited, which leads to the stabilization of HIF-1*α* and its accumulation in the nucleus [14, 17]. All P4Hs are 2-oxoglutarate dioxygenases and require Fe^2+^, 2-oxoglutarate, O_2_, and ascorbate. Thus, even small decreases in the O_2_ concentration will inhibit the activities of the HIF-P4Hs so that HIF-1*α* escape degradation [17].

Recent evidences suggested that hypoxia is involved in the chondrogenic differentiation of MSCs [18]. Expansion and chondrogenic induction of the bone marrow-derived MSCs under hypoxia generally result in enhanced chondrogenic differentiation, while they showed a reduced capacity in differentiating into adipogenic and osteogenic lineage [19–21].

In in vitro studies, hypoxia is generally induced by decreasing atmospheric oxygen concentrations or by utilization of mimetic chemical agents such as cobalt chloride (CoCl_2_) and desferrioxamine (DFO). Chemical agents are more attractive in experimental laboratories because they are cheap, they maintain steady oxygen tension, and they are more stable compared to hypoxic chamber. They artificially induce hypoxia through blocking the degradation of HIF-1*α* [22]. Cobalt chloride has been reported to inhibit the activities of HIF-P4Hs and FIH, suggesting that it may occupy their Fe^2+^ binding site and block the degradation of HIF-1*α* [17]. It is demonstrated that the effects of hypoxia-mimetic agents are comparable to those resulting from reduced atmospheric oxygen levels [17].

Despite several studies on the impact of hypoxia preconditioning on MSC differentiation, the influence of hypoxia on MSC behavior is still a matter of discussion. The data on their response to hypoxic conditions are rather controversial, demonstrating both damaging and ameliorating effects. The present study was aimed to compare the effects of hypoxia, induced by CoCl_2_, among the human MSCs derived from human dental pulp, adipose tissue, and Wharton's jelly umbilical cord, and their response to chondrogenic induction under hypoxic conditions.

## 2. Materials and Methods

### 2.1. Mesenchymal Stem Cells

The human adipose mesenchymal stem cells (ADMSCs) were purchased from Invitrogen (Life Technologies, Monza, Italy). The human dental pulp mesenchymal stem cells (DPMSCs) were obtained from healthy permanent premolars extracted during orthodontic treatment, under informed consent [9]. The human umbilical cord mesenchymal stem cells (UBMSCs) were obtained from the tissue of umbilical cords of full-term pregnancies. Informed consent was obtained from each patient according to the guidelines of the National Bioethics Committee, and the samples were treated following a protocol approved by the University of Bologna. All the cells were maintained at 37°C and 5% CO_2_ in DMEM/F12 Glutamax® medium (Gibco, Thermo Fisher, Monza, Italy) supplemented with 10% FBS (*v*/*v*) and 1% (*v*/*v*) penicillin and streptomycin (Gibco, Thermo Fisher, Monza, Italy). In this study, all the cells were used between passages 4 and 7.

### 2.2. Immunophenotyping

The DPMSCs, UCMSCs, and ADMSCs were checked for their surface marker profile by FACSCalibur flow cytometry system (Becton Dickinson, CA, USA) as already described [8, 9, 23]. Briefly, the MSCs were detached from the surface of the flask with enzyme digestion for 3 minutes at room temperature, collected, and centrifuged at 300*g* for 5 minutes. The pellets were resuspended in stain buffer and the cells were counted by hemocytometer. Then, 2.5 × 10^5^ cells were incubated for 45 min, in the dark at 4°C, with the following antibodies: fluorescein isothiocyanate- (FITC-) labeled mouse antihuman CD90 (StemCell Technologies, Milan, Italy), CD105, CD14, and CD19 (Diaclone, France); R-phycoerythrin- (PE-) labeled mouse antihuman CD34, CD44, CD45 (Diaclone, France), and CD73 (Becton Dickinson, CA, USA); and anti-HLA-DR (Diaclone, France). The control for FITC- or PE-coupled antibodies was isotypic mouse IgG1. The data were evaluated using CellQuest software (Becton Dickinson, CA, USA).

### 2.3. Cobalt Chloride Treatment and MTT Assay

The cells were seeded in 96-well culture plates at a density of 1 × 10^4^ cells/well for 24 h. The culture medium was changed to fresh MEM containing 2% FBS and 1% antibiotics and treated with different concentrations of CoCl_2_ ranging from 50 to 400 *μ*M. After 24 h and 48 h, the medium was changed with a new one supplemented with 0.5 mg/mL of 3-(4,5-dimethylthiazol-2-yl)-2,5-diphenyltetrazolium bromide (MTT) for 3 h at 37°C. The formazan produced was dissolved by solvent solution (0.1 N HCl in isopropanol), and the optical density was read at 570 nm by microplate reader (Model 680, Bio-Rad Lab Inc., CA, USA).

### 2.4. HIF-1*α* Protein Expression

The MSCs were treated with 100 *μ*M CoCl_2_ for 6 h, 12 h, 24 h, and 48 h, and cytosolic extract was obtained by using RIPA lysis buffer (Pierce, Thermo Fisher Scientific, Monza, Italy) supplemented with 25 *μ*mol/L protease inhibitor cocktail (Pierce, Thermo Fisher Scientific, Monza, Italy) and 1 *μ*L of *β*-mercapto-ethanol (Sigma-Aldrich, St. Louis, Missouri, USA). Total proteins were resolved on 4–12% SDS polyacrylamide gel electrophoresis (SDS–PAGE) and electrophoretically transferred into a nitrocellulose membrane using a wet blotting apparatus (Invitrogen, Thermo Fisher Scientific, Monza, Italy). The membranes were blocked with dry milk (Invitrogen, Thermo Fisher Scientific, Monza, Italy) for 30 minutes at room temperature and were then incubated with antihuman HIF-1*α*, diluted 1 : 400 (Invitrogen, Thermo Fisher Scientific, Monza, Italy), and antihuman actin, diluted 1 : 1000 (Cell Signaling Technology, Leiden, The Netherlands) at 4°C over night. After washing with transfer buffer (TBS-T), each blot was incubated with antirabbit secondary antibody (1 : 5,000 dilution; Cell Signaling Technology, Leiden, The Netherlands) for 1 h and 30 minutes at room temperature. The antibody signal was visualized with the enhancement chemiluminescence system (Pierce, Thermo Fisher Scientific, Monza, Italy). Images were obtained by using Image Station 2000R (Kodak, NY, USA).

Band densitometry was measured using ImageJ software (National Institutes of Health), and the intensities of the specific protein bands were corrected for equal actin loading; they were expressed as relative to the intensity of the control sample. Data showed the average of triplicates ± SD and were representative from three independent experiments.

### 2.5. Chondrogenic Differentiation

The MSCs, in micromass culture, were stimulated with MEM supplemented with 2% FBS and 100 *μ*M CoCl_2_ for 48 h and then were cultured in chondrogenic medium consisting in MEM supplemented with 2% FBS, 10 ng/mL of TGF-*β*3 (Millipore, Milan, Italy), 100 nm dexamethasone (Sigma-Aldrich, St. Louis, Missouri, USA), 100 *μ*g/mL ascorbate-2-phosphate (Sigma-Aldrich, St. Louis, Missouri, USA), ITS (6.25 *μ*g/mL insulin, 6.25 *μ*g/mL transferrin, 6.25 *μ*g/mL selenous acid) (Gibco, Thermo Fisher Scientific, Monza, Italy) for 7, 14, 21, and 28 days. The chondrogenic medium was replaced every 3 days. Control samples consisted in MSCs cultured in micromass system in MEM supplemented with 10% FBS up to 28 days.

At the end of each treatment, the cells were collected and RNA extraction and quantitative real time PCR (qRT-PCR) was performed.

### 2.6. mRNA Extraction and qRT-PCR

RNeasy Mini Kit (Invitrogen, Thermo Fisher Scientific, Monza, Italy) was used for the extraction of RNA from cellular pellets, and 200 ng of total RNA was reverse transcribed into first-strand cDNA using SuperScript™ III One-Step RT-PCR System (Invitrogen, Thermo Fisher Scientific, Monza, Italy). Chondrogenic mRNA marker expression levels were analyzed via real-time PCR by 7500 real-time PCR machine (Applied Biosystems, Life Technologies, Monza, Italy). For mRNA quantification, TaqMan assays (Life Technologies, Thermo Fischer Scientific, Monza Italy) were used specific for collagen type II (COL2A1; Hs00264051_m1), collagen type 10 (COL10A1; Hs00166657_m1), Sox9 (SOX9; Hs01001343_g1), versican (VCAN; Hs00171642_m1), and aggrecan (ACAN; Hs00153936_m1). Relative gene expression levels were normalized to that of glyceraldehyde 3-phosphate dehydrogenase (GAPDH; Hs99999905_m1). Data are presented as fold changes relative to levels in control samples by using formula 2^−ΔΔ*CT*^, as recommended by the manufacturer (User Bulletin number 2 P/N 4303859; Applied Biosystems).

### 2.7. Alcian Blue and Safranin O Staining

The MSCs were stimulated with MEM supplemented with 2% FBS and 100 *μ*M CoCl_2_ for 48 h in micromass culture, and then they were cultured in chondrogenic medium as previously described. At the end of each experimental point, the MSCs were immediately fixed in 4% formaldehyde (Sigma-Aldrich, St. Louis, Missouri, USA) in phosphate buffer (PBS) for 24 h at 4°C. Then, they were dehydrated in a graded series of ethanol and embedded in paraffin wax (Fluka, Sigma-Aldrich). Paraffin sections of 6 *μ*m were obtained with an automated rotary microtome (Leica Microsystems Srl, Cambridge, United Kingdom) and collected on Superfrost glass slides (Carl Roth, Karlshure, Germany). Samples were subsequently processed for alcian blue staining by using alcian blue and safranin O staining kits (Bio-Optica, Milan, Italy). Images utilized are representative from three independent experiments.

### 2.8. Statistical Analysis

MTT and real-time PCR values were presented as the mean ± standard deviation, and each type of experiment was replicated three times. One way ANOVA followed by Dunnett's multiple comparison test was used to evaluate the differences between the samples. Statistical analysis was performed by GraphPad Prism 5.0 software (GraphPad Software Inc., San Diego, CA, USA). *P* values of <0.05 were considered statistically significant.

## 3. Results

### 3.1. Immunophenotyping

The MSCs utilized for all the experiments were characterized for CD105, CD14, CD19, CD34, CD45, CD73, CD90, and HLADR using flow cytometric analysis. It was found that these cells were highly positive for CD105, CD73, and CD90 (>95%) and negative for CD34, CD19, CD45, CD14, and HLA-DR (<3%) (data not showed) [8, 9, 23]. Moreover, they were able to differentiate into all the mesenchymal lineages (data not showed) [8, 9, 23].

### 3.2. MTT Assay

**Figure 1:**
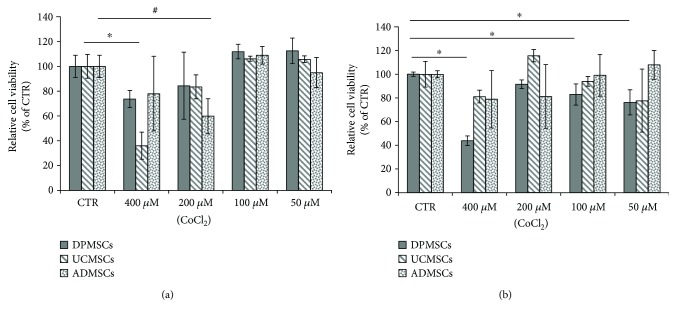
Effects of different concentration of CoCl_2_ on the DPMSCs, UCMSCs, and ADMSCs. (a) Cells treated for 24 h; (b) cells treated for 48 h. Cell viability was determined by MTT assay and the results were expressed as relative cell viability compared to each control sample. Each value is the mean ± SD of triplicate independent experiments. ^∗^*p* < 0.05, as compared to control DPMSCs; ^#^*p* < 0.05, as compared to control UCMSCs; ^§^*p* < 0.05, as compared to control ADMSCs.

To evaluate the potential toxicity of CoCl_2_ on MSCs cells, an MTT assay testing different concentration of CoCl_2_, ranging from 50 *μ*Μ to 400 *μ*M, was carried out, for 24 h and 48 h. Results showed a high cell viability in all the three MSCs tested for 24 h and 48 h (Figures 1(a) and 1(b)). A light reduction of viability was observed after 48 h with the concentration of 400 *μ*M (Figure 1(b)). The concentration of 100 *μ*M showed the highest cell viability both at 24 h and 48 h; therefore, this concentration was used for all the following experiments.

### 3.3. HIF-1*α* Expression

**Figure 2:**
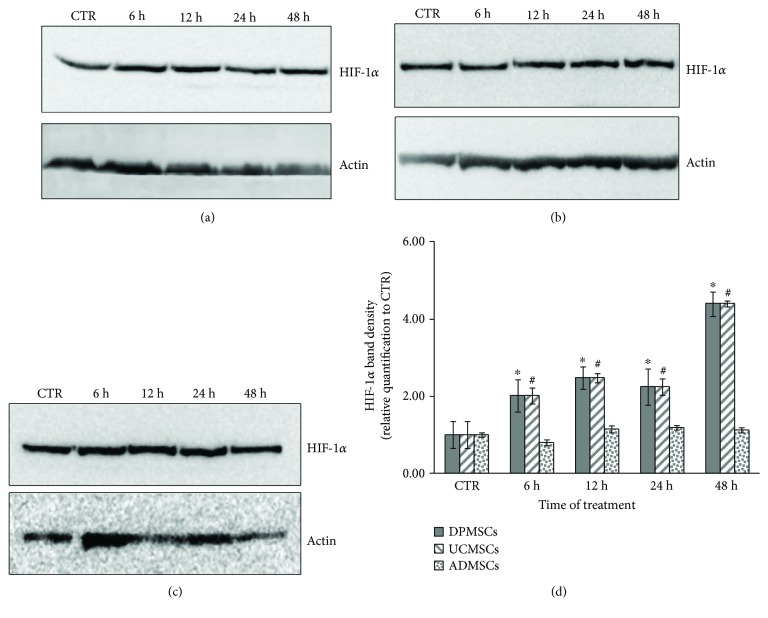
Effect of CoCl_2_ in the expression of HIF-1*α*. The MSCs were incubated with 100 *μ*M of CoCl_2_ in cell medium supplemented with 2% of FCS for 6 h, 12 h, 24 h, and 48 h. (a) HIF-1*α* expression in the DPMSCs; (b) HIF-1*α* expression in the UCMSCs; (c) HIF-1*α* expression in the ADMSCs; (d) densitometric analysis of western blot bands. Control samples consist in the MSCs cultured with cell medium supplemented with 10% FCS for 48 h. The value was normalized to each corresponding *β*-actin level and represented as a relative expression compared to the control sample. Each value is the mean ± SD of triplicate independent experiments. ^∗^*p* < 0.05, as compared to control DPMSCs; ^#^*p* < 0.05, as compared to control UCMSCs; ^§^*p* < 0.05, as compared to control ADMSCs.

To determine if the treatment with CoCl_2_ was responsible of an upregulation of the transcription factor HIF1*α*, the expression of the protein after 6 h, 12 h, 24 h, and 48 h of CoCl_2_ exposition was evaluated by western blotting analysis (Figure 2).

Apparently, no difference between control and treated samples was observed by western blot, in the entire MSCs tested (Figures 2(a)–2(c)). Densitometric analysis of protein bands showed a time-dependent upregulation of HIF-1*α* in DPMSCs and UCMSCs, while no significant difference was evaluated in ADMSCs (Figure 2(d)). After 48 h of CoCl_2_ incubation, the expression of HIF-1*α* was increased at about 4.4-fold in DPMSCs and UCMSCs (Figure 2(d)) compared to that in each control sample.

### 3.4. Chondrogenic Differentiation of DPMSCs, UCMSCs, and ADMSCs in Hypoxia

**Figure 3:**
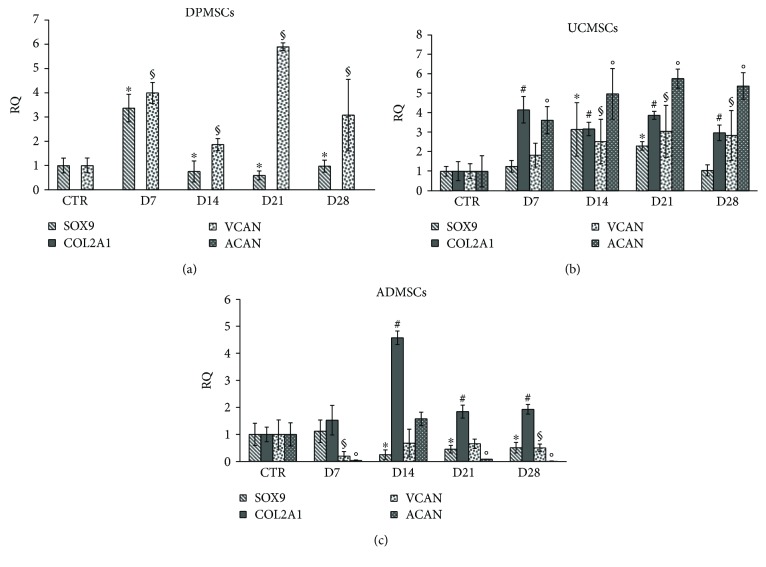
Effects of CoCl_2_ on the MSCs induced to chondrogenic phenotype for 7, 14, 21, and 28 days. mRNA expression of SOX9, COL2A1, VCAN, and ACAN in (a) DPMSCs, (b) UCMSCs, and (c) ADMSCs. Gene expression was normalized to the corresponding GAPDH and calculated as relative expression compared to control cells for the DPMSCs and UCMSCs and to D14 for the ADMSCs. The experiments were performed three times. Data were expressed as mean ± SD. ^∗^*p* < 0.05, as compared to control SOX9 mRNA; ^#^*p* < 0.05, as compared to control COL2A1 mRNA; ^§^*p* < 0.05, as compared to control VCAN; °*p* < 0.05, as compared to control ACAN.

To determine if hypoxia, induced by CoCl_2,_ enhances chondrogenic differentiation, DPMSCs, UCMSCs, and ADMSCs were preincubated with 100 *μ*M CoCl_2_ for 48 h, and then they were induced to chondrogenic differentiation by chondrogenic medium up to 28 days. At each experimental point, the gene expression of the chondrogenic markers SRY-box containing gene 9 (SOX9), a key transcription factor for chondrocyte differentiation, type II collagen (COL2A1), versican (VCAN), and aggrecan (ACAN), downstream targets of SOX9, was assessed by qRT-PCR (Figure 3).

The DPMSCs showed an upregulation of about 3-fold of the transcription factor SOX9 after 7 days of chondrogenic differentiation, followed by a reduction of expression after 14, 21, and 28 days of induction (Figure 3(a)). An upregulation of VCAN was also observed after 7, 14, 21, and 28 days of chondrogenic stimulation (Figure 3(a)), while no amplification was detected for mRNA corresponding to COL2A1 and ACAN (Figure 3(a)).

The UCMSCs showed a time-dependent upregulation of all the chondrogenic markers tested (Figure 3(b)). The SOX9 mRNA level reached the highest level after 21 days of differentiation, while COL2A1 mRNA expression was observed after 14 days of differentiation and increased about 5-fold after 28 days (Figure 3(b)). VCAN mRNA showed a light time-dependent upregulation, while the expression of ACAN mRNA was significantly increased up to 21 and 28 days of chondrogenic differentiation (Figure 3(b)).

On the contrary, the ADMSCs showed a constant expression of SOX9 and ACAN compared to control samples (Figure 3(c)), while an upregulation of expression was detected for COL2A1 and ACAN mRNA at the end of the chondrogenic induction (Figure 3(c)).

**Figure 4:**
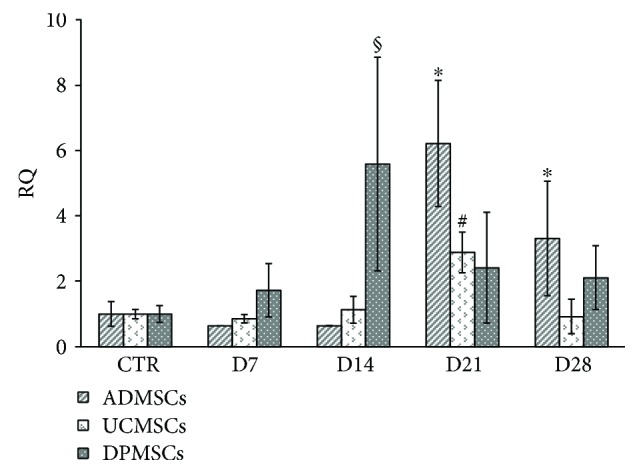
mRNA expression of COL10A in the ADMSCs, UCMSCs, and DPMSCs induced to chondrogenic differentiation up to 28 days. The experiments were performed three times. Data were expressed as mean ± SD. ^∗^*p* < 0.05, as compared to control ADMSCs; ^§^*p* < 0.05, as compared to control DPMSCs. ^#^*p* < 0.05, as compared to control UCMSCs.

To demonstrate the absence of hypertrophic chondrogenesis, a qRT-PCR to evaluate the expression of the hypertrophic chondrogenic marker, collagen type 10, was carried out. Results showed a low signal of collagen type 10 in the UCMSCs and DPMSCs after 28 days of differentiation (Figure 4), while the ADMSCs showed a light but statistically significant upregulation of collagen type 10 compared to the control samples (Figure 4).

### 3.5. Alcian Blue and Safranin O Staining

**Figure 5:**
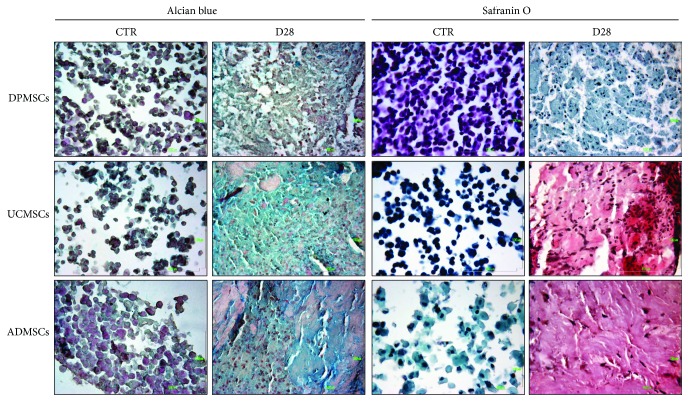
Alcian blue and safranin O staining on MSC micromasses, previously treated with 100 *μ*M CoCl_2_ and subsequently stimulated to chondrogenic differentiation up to 28 days. The images are representative of three different experiments (magnification 400x; bar: 100 *μ*M).

To demonstrate the presence of proteoglycans in the extracellular matrix produced by the MSCs stimulated to chondrogenic differentiation up to 28 days, alcian blue and safranin O stainings were carried out. Alcian blue results showed an intense blue color on extracellular matrix produced by the UCMSCs and ADMSCs (Figure 5), while a very light signal was detected in the DPMSCs. These data are supported by safranin O staining, which demonstrated a clear red color, corresponding to proteoglycan deposition, in the UCMSCs and ADMSCs, while no red signal was detected in the DPMSCs (Figure 5).

## 4. Discussion

Articular cartilage damage caused by sports injuries, accidental trauma, and aging generally progresses to more serious joint disorders, including osteoarthritis (OA), necrosis of subchondral bone, or arthritis [1, 2]. The hyaline articular cartilage has a very limited or no intrinsic capacity for repair, and even minor lesions or injuries may trigger progressive damage and joint degeneration [1]. Current treatments, based on surgical interventions, are still not satisfactory, often followed by the development of fibrocartilage [2]. Nowadays, repair and regeneration of hyaline cartilage are still a challenge. Novel cell-based tissue engineering techniques have been proposed with the aim to repair defects with bioengineered tissue that mimics the properties of hyaline cartilage and helps in the integration into native tissue. Transplantation of MSCs is a promising strategy based on their high proliferative capacity, self-renewal, and potential to differentiate into cartilage-producing cells. However, still biological obstacles persist in the MSC-based regeneration of articular cartilage [1]. Mechanical properties of the reconstructed cartilage are inferior to the native tissue, and heterogeneity of initial cell population and poor matrix deposition contribute to functional limitation of the MSCs [1].

Increasing evidence indicates that environmental preconditioning is a powerful approach in promoting stem cell proliferation and chondrogenic potential [24]. In the native cartilage, cells are exposed to very low oxygen tension which promotes MSC survival, proliferation, and differentiation capacity [24]. However, the data regarding the effect of hypoxia on MSC behavior are still contradictory and the role of hypoxia is still unclear.

The aim of this study was to compare the behavior of three human MSCs, separated from dental pulp, Wharton's jelly umbilical cord, and adipose tissues, induced to chondrogenic phenotype under hypoxic environment.

The choice of DPMSCs, UCMSCs, and ADMSCs for this study reflects their readily accessible source, easy protocols for isolation, and easy availability without ethical concerns. They represent the ideal source for stem cell-based therapy and regenerative medicine. Although the DPMSCs, UCMSCs, and ADMSCs seem very similar, they show specific characteristic of the tissue of origin which diversifies their response to hypoxia and tissue regeneration.

Cobalt chloride, a chemical hypoxia-mimetic agent, is an attractive alternative to creating physical hypoxic agents [25–27]. The potential toxicity of CoCl_2_ on the different MSCs utilized in this study was investigated by MTT analysis. The highest cell viability was obtained at 100 *μ*M of CoCl_2_, after 24 h and 48 h in all the three MSCs tested. Therefore, 100 *μ*M was the concentration of cobalt chloride utilized in the study, in agreement with previous investigations on murine stem cells [21]. A pretreatment of 100 *μ*M CoCl_2_ was performed on DPMSCs, UCMSCs, and ADMSCs for 48 h, as a hypoxic preconditioning approach, with the aim to promote chondrogenic differentiation [24]. The mechanism by which hypoxia exerts its effect on cells is mainly regulated by HIF-1*α*, which upregulates several genes involved in glucose metabolism, erythropoiesis, iron transport, angiogenesis, and chondrogenesis [11, 13, 24]. To confirm the hypoxic condition induced by CoCl_2_, the presence of HIF-1*α* protein was evaluated. Results showed a time-dependent upregulation of HIF-1*α*, in the DPMSCs and UCMSCs, compared to untreated control, while no increase of expression of HIF-1*α* was detected in the ADMSCs, compared to control sample. Therefore, CoCl_2_ successfully mimicked the hypoxic condition in DPMSCs and UCMSCs but failed in inducing hypoxic microenviroment in ADMSCs. Our data are in agreement with previous results on murine bone marrow mesenchymal stem cells [21], human periodontal ligament MSCs [26], and DPMSCs [25] where the hypoxic induction by CoCl_2_ triggered the HIF-1*α* pathway.

The influence of hypoxia, induced by CoCl_2_, was subsequently investigated on the chondrogenic potential of DPMSCs, UCMSCs, and ADMSCs. The cell previously treated with CoCl_2_ for 48 h and then induced to chondrogenic differentiation up to 28 days showed different results regarding the mRNA expression of the chondrogenic markers analyzed. In the DPMSCs, while the transcription factor SOX9 and VCAN had an increase of expression, there was no expression of COL2A1 and ACAN. These findings suggest an inhibitory effect of hypoxia, on chondrogenic differentiation, in agreement with previous data on human periodontal ligament MSCs and human DPMSCs in which hypoxia induced by CoCl_2_ exposition inhibited osteogenic differentiation and enhanced the upregulation of the stem cell markers REX1 and OCT4, responsible for maintaining stemness [26].

Similar results were obtained in the ADMSCs, in which hypoxia induced by CoCl_2_ treatment induced a weak chondrogenic induction, as expected due to the lack of upregulation of HIF-1*α* protein observed by western blot. Few studies investigated the impact of hypoxia on differentiation of the ADMSCs yielded inconsistent and contrasting results [28]. It was found that oxygen tension at 2–5% significantly affected the differentiation capacity of ADMSCs, while oxygen tension at 1 and 1.5% maintained adipogenic, osteogenic, and chondrogenic differentiation [29]. Fotia and colleagues [29] reported that hypoxia enhances ADMSC proliferation and maintains the multipotency status, allowing the differentiation in specific lineages in the presence of proper factors. However, they demonstrated chondrogenic differentiation by alcian blue staining without any quantitative data on the mRNA expression of the chondrogenic markers. Other studies have demonstrated that low oxygen tension increased the ADMSC stemness marker expression and proliferation rate without altering their morphology and surface markers [30, 31]. Low oxygen tension further enhances the chondrogenic differentiation ability but reduces both adipogenic and osteogenic differentiation potential [32–34]. On the contrary, Pilgaard and colleagues [34] demonstrated that hypoxic pretreatment did not exhibit an enhanced chondrogenic differentiation in human ADMSCs, in agreement with our results. We hypothesize that higher concentration of CoCl_2_ or a longer duration of CoCl_2_ supplementation might be required to induce hypoxic effect on ADMSCs.

In our study, the UCMSCs demonstrated the best chondrogenic phenotype after a hypoxic pretreatment and chondrogenic induction up to 28 days. All the chondrogenic markers showed an upregulation of their expression compared to untreated control samples, supported by alcian blue and safranin O stainings, and a high expression of the transcription factor HIF-1*α*, responsible for the upregulation of the downstream chondrogenic genes. Previous studies demonstrated that chemical hypoxia, induced by 100 *μ*M CoCl_2_, induced proliferation and mitochondrial protection and did not alter the differentiation capacity of the human UCMSCs [35]. Reppel and colleagues [36] reported an increase in chondrogenic differentiation when Wharton's jelly-derived human MSCs were expanded under hypoxia, in agreement with our results.

## 5. Conclusion

Our data demonstrated that the effect of hypoxia on chondrogenic differentiation of MSCs was dependent on cell source. The UCMSCs are more prone to chondrogenic differentiation and to nonhypertrophic chondrogenesis, compared to the DPMSCs and ADMSCs, and these features could be correlated to the nature of the donor tissue and the different hypoxic environment of the umbilical cord compared to the adipose tissue and dental pulp [37–39]. These findings should be taken in great consideration in the choice of stem cells for prospective regenerative strategies.
